# Elevated Expression of Growth Differentiation Factor-15 Is Associated With Acute Exacerbation of Idiopathic Pulmonary Fibrosis

**DOI:** 10.3389/fimmu.2022.891448

**Published:** 2022-06-15

**Authors:** Mengshu Cao, Lina Gu, Lili Guo, Mengying Liu, Tianzhen Wang, Ji Zhang, Huizhe Zhang, Yufeng Zhang, Yanchen Shi, Yichao Zhao, Xiaohua Qiu, Xianhua Gui, Miao Ma, Yaqiong Tian, Xiaoqin Liu, Fanqing Meng, Yonglong Xiao, Lingyun Sun

**Affiliations:** ^1^Department of Respiratory and Critical Care Medicine, Nanjing Drum Tower Hospital, The Affiliated Hospital of Nanjing University Medical School, Nanjing, China; ^2^Department of Respiratory and Critical Care Medicine, Nanjing Drum Tower Hospital Clinical College of Traditional Chinese and Western Medicine, Nanjing University of Chinese Medicine, Nanjing, China; ^3^Department of Respiratory and Critical Care Medicine, Nanjing Drum Tower Hospital Clinical College of Nanjing Medical University, Nanjing, China; ^4^Department of Clinical Laboratory, Nanjing Drum Tower Hospital, The Affiliated Hospital of Nanjing University Medical School, Nanjing, China; ^5^Wuxi Transplant Center, Wuxi People’s Hospital Affiliated to Nanjing Medical University, Wuxi, China; ^6^Department of Pathology, Nanjing Drum Tower Hospital, The Affiliated Hospital of Nanjing University Medical School, Nanjing, China; ^7^Department of Rheumatology and Immunology, Nanjing Drum Tower Hospital, The Affiliated Hospital of Nanjing University Medical School, Nanjing, China

**Keywords:** growth differentiation factor 15, idiopathic pulmonary fibrosis, acute exacerbation, inflammation, metabolism

## Abstract

**Backgrounds:**

Growth differentiation factor 15 (GDF-15) is a highly divergent member of the TGF-β superfamily and has been implicated in various biological functions. However, the expression of GDF-15 in patients with acute exacerbation of idiopathic pulmonary fibrosis (AE-IPF) is unclear.

**Method:**

The study included 47 AE-IPF patients, 61 stable IPF (S-IPF) subjects, and 31 healthy controls (HCs). Serum GDF-15 levels and their expression in the lung were measured. The correlation between serum GDF-15 and other clinical parameters and the risk factors for AE occurrence and the survival of IPF patients were analyzed.

**Results:**

Serum GDF-15 levels were significantly elevated in AE-IPF patients (1279.22 ± 540.02 pg/ml) as compared with HCs (891.30 ± 479.90 pg/ml) or S-IPF subjects (107.82 ± 14.21 pg/ml) (both p < 0.001). The protein and mRNA expressions of GDF-15 in the lung of AE-IPF patients were significantly increased as compared with S-IPF cases (p = 0.007 and p = 0.026, respectively). The serum GDF-15 level was correlated with the clinical variables of inflammation, metabolism, and disease severity in IPF subjects (all p < 0.05). The GDF-15 serum concentration was significantly higher in decedents than in survivors (p = 0.005). A serum GDF-15 level above 989.3 pg/ml was a risk factor for AE occurrence (p = 0.04), and the level above 1,075.76 pg/ml was an independent predictor for survival in IPF cases (p = 0.007).

**Conclusions:**

The GDF-15 level was significantly elevated in subjects with AE-IPF. GDF-15 could be a promising biomarker for AE occurrence and survival in IPF patients.

## Introduction

Idiopathic pulmonary fibrosis (IPF) is the most common and devastating idiopathic interstitial lung disease (ILD), thus resulting in a progressive decline of respiratory function and early mortality (1). During the course of the disease, some cases may suffer an acute, clinically significant, respiratory deterioration with new widespread alveolar abnormality, referred to as acute exacerbation of IPF (AE-IPF) (2). The etiology of AE-IPF remains unclear. It may represent an intrinsic acceleration of the underlying fibrotic disease or a response to occult external events (e.g., infection, aspiration, and surgical operation) leading to acute lung injury (ALI) and histopathological diffuse alveolar damage (DAD), which shares the similar clinical and pathophysiological features with acute respiratory distress syndrome (ARDS) (3). AE is the primary cause of death in patients with this disorder (4). The incidence of AE is ranged from 7% to 32%, and nearly 46% of deaths in IPF patients are associated with AE in recent studies (1) and in-hospital mortality after AE exceeds 50% (5–7). Therefore, the incidences and predictors of AE are very important for clinicians who attempt to manage AE-IPF.

Growth differentiation factor 15 (GDF-15) is a divergent member of the transforming growth factor-β (TGF-β) superfamily, also named macrophage inhibitory cytokine-1 (MIC-1) (8). It is usually produced under stress conditions and has been involved in multiple biological processes, such as energy homeostasis, body weight regulation, and cachexia driven by cancer and chronic diseases (9). It has been linked to several acute and chronic pulmonary conditions, such as lung infection, chronic obstructive pulmonary disease (COPD), lung cancer, pulmonary embolism, pulmonary hypertension, and pulmonary fibrosis (9–12). From the data of lung genomics, GDF-15 is upregulated and expressed by epithelial cells in IPF. It is a useful biomarker of epithelial stress and can predict the poor outcomes in IPF patients (13). The association between aging and IPF is well established; GDF-15, the aging-related biomarker, has some of association with age, interstitial lung abnormalities, and mortality (14, 15). GDF-15 levels were elevated in critical coronavirus disease 2019 (COVID-19) patients and not correlated with the patient’s age or body mass index (BMI) (16). However, circulating levels of GDF-15 can predict the in-hospital mortality in COVID-19 patients from the recent study (17). So, GDF-15 derived from epithelial cells may be involved in the process caused by multiple etiologies.

For patients with AE-IPF, diffuse ground glass opacity (GGO) and consolidation on chest imaging and DAD on histopathology, similar to what is observed in ARDS subjects. However, the expression of GDF-15 in the acute exacerbation of IPF remains unknown. In present study, we aimed to explore the expression and potential role of GDF-15 in a Chinese cohort of AE-IPF subjects.

## Materials and Methods

### Study Population

The current study recruited 108 IPF subjects (stable IPF, n = 47 and AE-IPF, n = 61) and 31 healthy controls (HCs) in Nanjing Drum Tower Hospital, Nanjing University Medical School from January 2015 to December 2018. The diagnoses criteria of IPF were based on the updated international practical guideline in 2018 (18) and AE-IPF was according to the international working group report in 2016 (2). Stable IPF (S-IPF) is referred to the clinical symptoms, chest imaging, and pulmonary function tests being stable at least 1 month before the samples of peripheral blood are obtained (19). Lung specimens were collected through surgical lung biopsy (SLB) or lung transplantation from the Nanjing Drum Tower Hospital and Wuxi People’s Hospital (lung cancer patients, normal lung tissues adjacent to cancer, n=4; S-IPF subjects, n=4 and AE-IPF cases, n=4). Clinical data were collected from electronic medical records on admission and by a telephone follow-up. The current study was approved by the Ethics Committee of Nanjing Drum Tower Hospital in terms of the Declaration of Helsinki (1989) (NO.2016-160-01). All subjects signed the informed consent paperwork.

### Assay of Serum GDF-15

The serum samples were collected, aliquoted, and stored at -80 °C. The date of sample collection was the day following admission (S-IPF patients who were admitted for diagnoses or evaluating on conditions). The serum concentrations of GDF-15 (DY957; R&D Systems, Minneapolis, United States) and leptin (ab108879; Millipore Corporation, Massachusetts, United States) were measured using enzyme-linked immunosorbent assay (ELISA) kits on the basis of the manufacturer’s instructions. Each sample was assayed in duplicate.

## GDF-15 Measurement in Lung Tissues

Quantitative real-time PCR (RT-PCR) and western blot (WB) were used to detect the mRNA and protein expression of GDF-15 in the lung from IPF patients and HCs as described previously (20). Immunohistochemistry (IHC) was employed to determine the location of GDF-15 expression in the lung. Total RNA was isolated from frozen lung tissues with a Trizol reagent (15596026 and 15596018) (Invitrogen, California, United States). The primers is right were designed and synthesized by TaKaRa Biotechnology Co, Ltd. (Dalian, China). The primers of human GDF-15 were forward GACCCTCAGAGTTGCACTCC and reverse GCCTGGTTAGCAGGTCCTC, and for GAPDH, they were forward GGAGCGAGATCCCTCCAAAAT and reverse GGCTGTTGTCATACTTCTCATGG, respectively. The anti-GDF-15 (ab189358; Abcam, Boston, United States) and GAPDH (14C10, CST, Boston, United States) were used in WB and anti-GDF-15 (NBP1-81050; Novus Biologicals, Colorado, United States) was employed in IHC as the primary antibodies. Goat anti-rabbit and rabbit anti-mouse secondary antibodies (7076S and 7074S; CST, Boston, United States) were used. The percentages of GDF-15 positive expressing cells in lung tissue were analyzed by software Image-Pro Plus (6.0 version).

### Collection of Clinical Data

Clinical data were collected from electronic medical records. The vital status was determined from medical records or a telephone follow-up. The deadline of the follow-up was November 17, 2019. The duration of survival was from the date of sample collection to the date of death or vital status confirmed. Concurrent infection was defined as patients with clinical symptoms of fever or/and purulent sputum, elevated white blood cell (WBC) counts, C-reactive protein (CRP), erythrocyte sedimentation rate (ESR), and/or lactate dehydrogenase (LDH), or positive pathogen tests.

### Assessment of Disease Severity

The scores of gender–age–physiology (GAP) and chest high-resolution computed tomography (HRCT) were used to assess the disease severity of IPF patients. The total GAP score was calculated by the method proposed by Ley et al. (21). Chest HRCT was performed with 1.0–1.5 mm thick sections and appropriate window settings (window width: 1,600, window level: -600). The images were evaluated for the extents of GGO, consolidation, reticulation, traction bronchiectasis, honeycombing, and emphysema. The chest HRCT scores were assessed according to the published study by Lynch et al. (22).

### Statistical Analysis

The categorical variables were presented as numbers and percentages, and continuous variables were expressed as the mean ± standard deviation (SD). The comparisons of the differences of categorical parameters were assessed by the chi-square (χ^2^) or Fisher exact test, and continuous variables were evaluated by independent sample *t*-test, Kruskal–Wallis, Mann–Whitney U test, or one-way ANOVA test. The correlations between serum GDF-15 and other clinical variables were assessed by Spearman’s or Pearson’s correlation analysis depending on the distribution of the variables. The survival curves were generated by Kaplan–Meier methods and compared by the log-rank test. The optimal cut-off value for predicting AE occurrence and the mortality was determined by receiver operating characteristic (ROC) curve. Logistic models were used to analyze the risk factors for AE and Cox regression models were built to examine the predictors of time-to-death in IPF patients. p < 0.05 was considered to be statistically significant. Statistical analyses were performed by IBM SPSS version 21 (SPSS, Inc., Chicago IL, United States) and Prism version 8 (GraphPad, San Diego, CA, United States).

## Results

### Baseline Characteristics in HCs, S-IPF, and AE-IPF Patients

As shown in **Supplementary Table 1**, the differences of gender and age in the three groups (HCs, S-IPF, and AE-IPF groups) were not significant (p = 0.794 and p = 0.085, respectively). However, there were significant differences in GDF-15 serum levels, WBC counts, triglyceride (TG), total cholesterol (TCHL), low cholesterol (LCHL), and glucose (GLU), which are related to infection and metabolism (p < 0.001, p < 0.001, p = 0.003, p = 0.014, p = 0.022, and p < 0.001, respectively). The differences of complication and prior antifibrotic use between patients with S-IPF and AE-IPF were not significant (p = 0.438 and p = 0.438, respectively).

### The Differences of Clinical Parameters Related to Infection and Inflammation, Metabolism, and Disease Severity Were Significant Between S-IPF and AE-IPF Patients

Between S-IPF and AE-IPF subjects, we compared the differences in the clinical variables of demography, inflammation, metabolism, and disease severity. The differences of gender, age, and smoking in patients between the two groups were not significant (p *=* 0.837, p = 0.308, and p = 0.971, respectively) (**Table 1**). Among the numbers of clinical variables associated with inflammation and infection, cases with prior corticosteroid use and fever in the AE-IPF group were significantly more than those in the S-IPF group (p = 0.002 and p < 0.001, respectively). In AE-IPF subjects, the CD4+ lymphocyte counts were significantly lower (p < 0.001), while the inflammatory indicators of WBC counts, CRP, ESR, and LDH were significantly increased compared with S-IPF cases (all p < 0.001). The clinical variables associated with metabolism, including TG, Apo AI, and AIB were significantly lower (p = 0.008, p = 0.023, and p < 0.001, respectively) and GLU was significantly higher in the AE-IPF group than those in the S-IPF group (p < 0.001). The clinical parameters related to the disease severity of IPF, including the PaO_2_/FiO_2_ ratio, D-dimer, HRCT score, pulmonary arterial pressure (PAH), forced vital capacity (FVC), carbon monoxide–diffusing capacity (DLCO), GAP scores, and stages were significantly worse in the AE-IPF group compared with the counters (all p < 0.05) (**Table 1**).

**Table 1:** Comparison of clinical features between S-IPF and AE-IPF patients.

Clinical variables	S-IPF group	AE-IPF group	p-value
**Population demography**			
Gender (M/F)	49/12	37/10	0.837
Age (years)	67.0 ± 8.3	68.5 ± 7.2	0.308
Smoking (pack*years)	16.8 ± 21.5	16.9 ± 22.0	0.971
**Inflammation and infection**			
Concurrent infection	9/52	26/21	<0.001
Prior corticosteroid use (Y/N)	27/34	35/12	0.002
Fever (Y/N)	3/58	19/28	<0.001
CD4+ lymphocyte counts (×10^9^)	0.703 ± 0.281	0.352 ± 0.302	<0.001
WBC counts (×10^9^)	6.50 (2.6-11.3)	10 (2.7-20.1)	<0.001
CRP (mg/L)	3.50 (1.0-126.5)	17.5 (1.0-442.6)	<0.001
ESR (mm/h)	15 (0-84)	38 (2-94)	<0.001
LDH (U/L)	231 (166-407)	389 (181-1267)	<0.001
**Metabolism**			
TG (mmol/L)	1.56 ± 1.12	1.09 ± 0.85	0.008
Apo AI (g/L)	1.10 ± 0.30	0.97 ± 0.28	0.023
AIB (g/L)	39.36 ± 4.09	33.13 ± 5.58	<0.001
GLU (mmol/L)	4.86 (3.53-13.84)	5.83 (2.03-17.67)	<0.001
**Disease severity**			
PaO_2_/FiO_2_ ratio	357 (224-476)	195 (49.0-496.0)	<0.001
D-dimer (mg/L)	0.35 (0.11-2.52)	1.05 (0.21-28.96)	<0.001
HRCT Score	3.45 ± 1.27	6.57 ± 1.16	<0.001
PAH (<40/≥40 mmHg)	44/4	14/18	<0.001
FVC (L)	2.33 ± 0.83 (59)	1.76 ± 0.53 (34)	0.001
DLCO % pred	55.95 ± 20.08 (54)	34.92 ± 19.53 (25)	<0.001
GAP scores	3.71 ± 1.67	5.39 ± 1.71	<0.001
GAP stage (I/II/III)	28/22/8	5/11/17	<0.001

### The Expressions of GDF-15 Were Significantly Elevated in AE-IPF Patients

**Figure 1:**
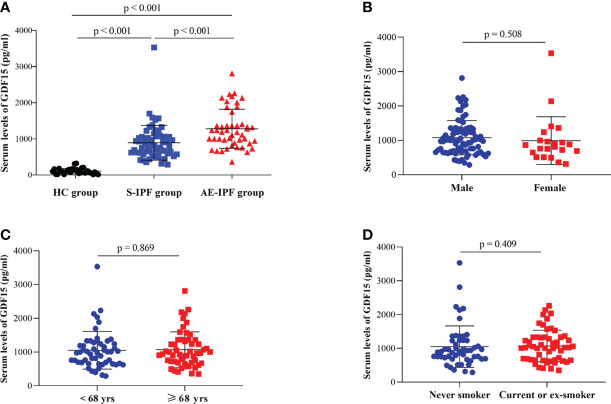
Serum levels of GDF-15 in IPF patients. **(A)** Serum GDF-15 concentrations were elevated significantly in AE-IPF patients compared with HCs and S-IPF cases (p *<* 0.001 and p *<* 0.001, respectively). Serum GDF-15 levels were also increased significantly in S-IPF subjects compared with HCs (p *<* 0.001). **(B–D)** The differences of serum GDF-15 levels were not significant in the different gender, age, and smoking history of IPF patients (p *=* 0.508, p *=* 0.869 and p *=* 0.409, respectively).

The serum level of GDF-15 in AE-IPF patients (1,279.22 ± 540.02 pg/ml) was significantly elevated compared with S-IPF subjects (891.30 ± 479.90 pg/ml) or HCs (107.82 ± 14.21 pg/ml) (p < 0.001 and p < 0.001, respectively) (**Figure 1A**). The differences of GDF-15 concentrations were not significant in IPF cases with different gender, age, and smoking history (p = 0.508, p = 0.869 and p = 0.409, respectively) (**Figures 1B–D**). The serum levels of both GDF-15 and leptin were increased (**Supplementary Figure 1A**). GDF-15 serum levels were significantly negatively correlated with leptin in AE-IPF subjects (r = - 0.329, p = 0.024) (**Supplementary Figure 1B**) but not in S-IPF or HC cases (r = 0.180, p = 0.168 and r = - 0.345, p = 0.062, respectively) (**Supplementary Figures 1C–D**).

**Figure 2:**
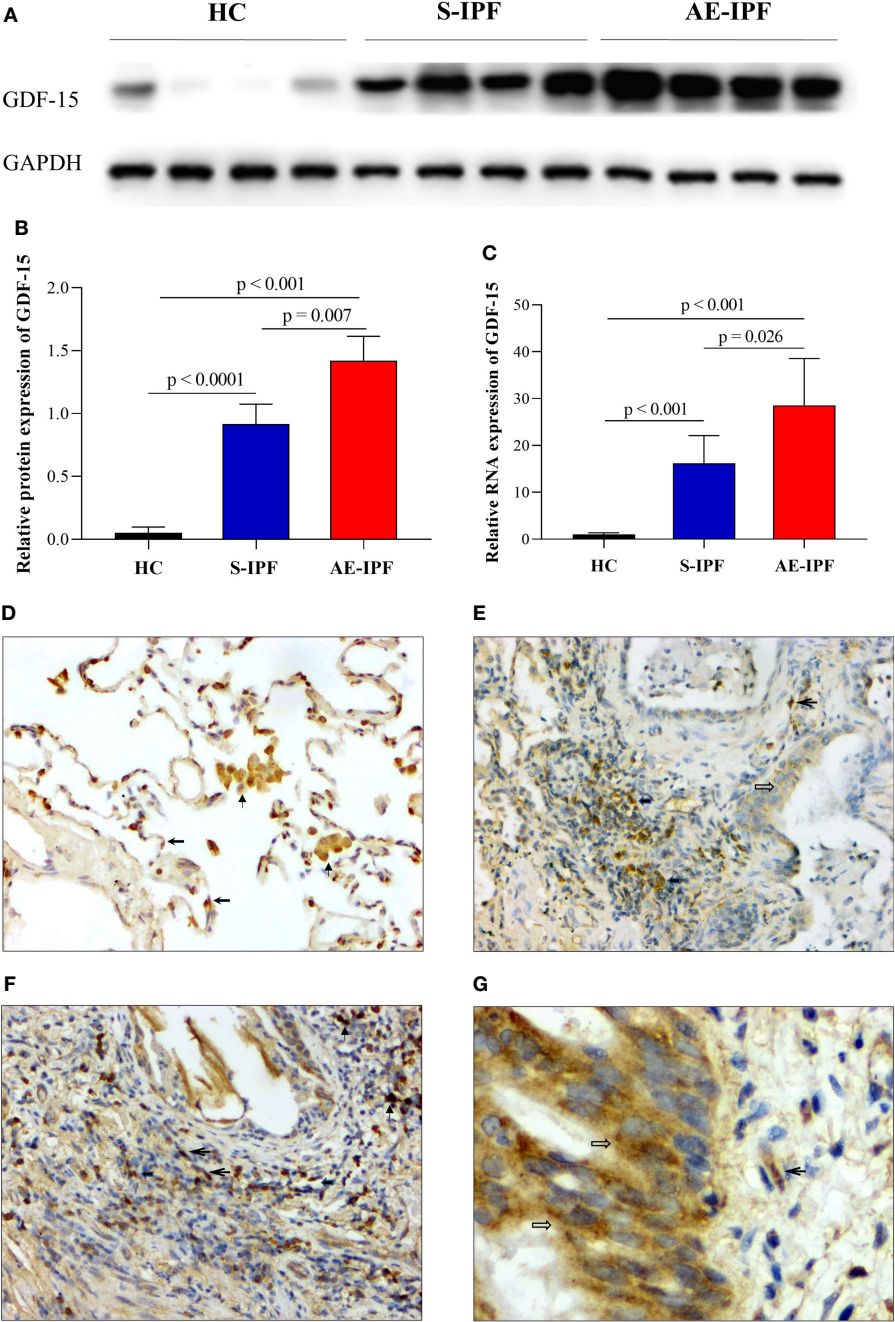
GDF-15 expression in the lung of IPF patients. **(A)** GDF-15 protein expression in the lung tissues of subjects with HC, S-IPF, and AE-IPF by WB. **(B)** The quantification of A showed that the protein expression of GDF-15 was significantly upregulated in AE-IPF patients compared with S-IPF subjects and HCs (p = 0.007 and p < 0.001, respectively). **(C)** GDF-15 mRNA expression in the lung of AE-IPF patients was significantly increased compared with HC, S-IPF cases by RT-PCR (p < 0.001 and p < 0.026, respectively) (n = 4, each group). **(D–G)** GDF-15 expression in the lung measured by IHC. **(D)** The moderately positive expressions of GDF-15 in the cytoplasm of type II AECs and macrophages in HC (IHC, ×200). **(E)** The moderately positive expressions of GDF-15 in inflammatory cells and macrophages in the alveolar septum and mild positive expressions in the bronchial epithelial cells and fibroblasts in S-IPF patients (IHC, ×200). **(F)** GDF-15 moderately positive expressions in inflammatory cells, macrophages, and fibroblasts in the interstitial spaces in AE-IPF case (IHC, ×200). **(G)** The moderately positive expressions of GDF-15 in the bronchial epithelial cells and fibroblasts in the same AE-IPF patient as **Figure 2F** (IHC, ×400). (Notes: macrophages 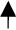; type II AEC 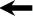; inflammatory cell 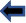; bronchial mucosa epithelial cells 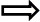; fibroblast 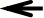).

The protein and mRNA expressions of GDF-15 in the lung of AE-IPF subjects were increased significantly compared with HCs and S-IPF cases by WB and RT-PCR (p < 0.001, p = 0.007 and p < 0.001, p = 0.026, respectively) (**Figures 2A–C**). Immunohistochemistry (IHC) staining showed the moderately positive expressions of GDF-15 in the cytoplasm of type II alveolar epithelial cells (AECs) and macrophages of HCs (**Figure 2D**, ×200), moderately positive expressions in inflammatory cells and macrophages in the alveolar septum and mild positive expression in the bronchial epithelial cells and fibroblasts of an S-IPF patient (**Figure 2E**, ×200), while there were moderately positive expressions in inflammatory cells, macrophages, and fibroblasts in the interstitial spaces (**Figure 2F**, ×200) and the bronchial epithelial cells of AE-IPF subjects (**Figure 2G**, ×400). The percentages of GDF-15- expressing cells in the lung of AE-IPF patients (23.67 ± 1.43%) were more than both HCs (15.34 ± 1.38%) and S-IPF (17.66 ± 1.10%) subjects (p *<* 0.001 and p < 0.001, respectively) (5 fields each sample).

### Serum GDF-15 Levels Correlated With the Clinical Variables of Inflammation, Metabolism, and Disease Severity in IPF Patients

As shown in **Table 2**, serum GDF-15 concentrations did not show any correlation with population demographic parameters, gender, age, and smoking status in all IPF patients. However, it was positively correlated with several clinical parameters of infection and inflammation (ESR, CRP, LDH, and PCT) (r = 0.314, p = 0.001, r = 0.376, p < 0.001, r = 0.272, p = 0.004 and r = 0.426, p = 0.01 respectively), and negatively correlated with metabolic indicators (TG, TC, LCHL, Apo B, and ALB) (r = - 0.201, p =0.037, r = - 0.229, p = 0.017, r = -0.255, p = 0.008, r = - 242, p = 0.011 and r = - 0.318, p = 0.001, respectively). Furthermore, serum GDF-15 levels were significantly correlated with the clinical variables of disease severity (D-dimer, PaO_2_/FiO_2_, HRCT score, PAH, FVC % pred, TLC % pred, DLCO % pred, and GAP stages (all p < 0.05).

**Table 2:** Bivariate correlation analysis between serum GDF-15 and clinical variables in IPF patients.

Clinical variables	r	p-value
**Population demography**		
Gender	-0.064	0.507
Age (years)	-0.021	0.832
Smoking (pack*years)	0.046	0.634
**Inflammation and infection**		
WBC counts (×10^9^)	0.125	0.196
ESR (mm/h)	0.314	0.001
CRP (mg/L)	0.376	<0.001
LDH (U/L)	0.272	0.004
PCT	0.426	0.01
**Metabolism**		
TG (mmol/L)	-0.201	0.037
TC (mmol/L)	-0.229	0.017
TCHL (mmol/L)	-0.100	0.302
LCHL (mmol/L)	-0.255	0.008
Apo AI (g/L)	-0.185	0.056
Apo B (g/L)	-0.242	0.011
ALB (g/L)	-0.318	0.001
GLU (mmol/L)	-0.018	0.850
**Disease severity**		
D-dimer (mg/L)	0.281	0.004
PaO2/FiO2 ratio	-0.297	0.003
HRCT score	0.330	0.001
PAH (mmHg)	0.322	0.004
FVC % pred	-0.222	0.033
TLC % pred	-0.325	0.003
DLCO % pred	-0.359	0.001
GAP stage (I/II+III)	0.305	0.003

### Serum GDF-15 Is a Risk Factor for AE Occurrence in IPF Patients

**Figure 3:**
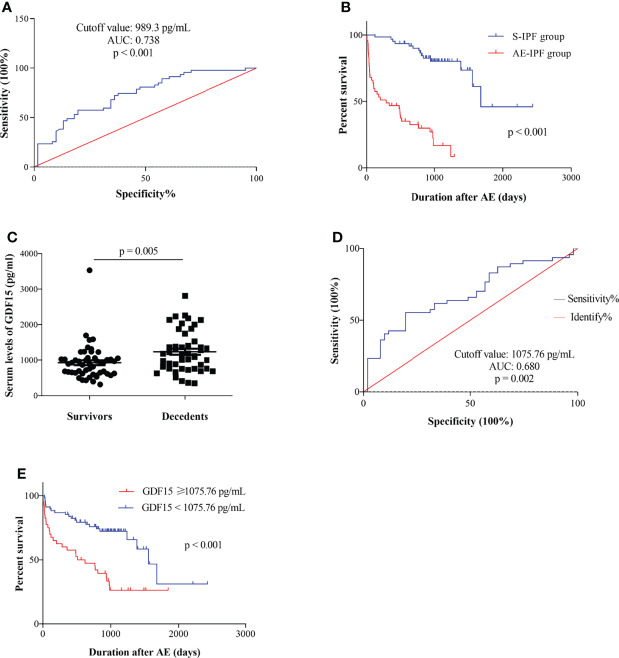
Serum GDF-15 can predict AE occurrence and survival in IPF patients independently. **(A)** The areas under ROC curves of GDF-15 were statistically significant in the classification of AE-IPF subjects (n = 47) or S-IPF patients (n = 61) (ROC: 0.738, p < 0.001, 95%CI: 0.529-0.809, cut-off value 989.3 pg/ml). **(B)** The AE-IPF patients had a significantly greater mortality compared with S-IPF cases by Kaplan–Meier analyses (p *<* 0.001). **(C)** Serum GDF-15 was significantly increased in decedents (n = 47) than that in survivors (n = 51) (p = 0.005). **(D)** ROC curve analyses for predicting the death of IPF patients based on serum GDF-15 levels. The areas under the ROC curve of GDF-15 were statistically significant in identifying the decedent from the survivor (ROC: 0.680, p = 0.002, 95%CI: 0.572-0.787, cut-off value 1075.76 pg/ml). **(E)** Patients with serum GDF-15 levels above 1,075.76 pg/ml had a higher mortality than those with GDF-15 levels lower than that (p *<* 0.001).

The areas under the ROC curves of serum GDF-15 levels were statistically significant in the classification of AE-IPF (n = 47) or S-IPF patients (n = 61) (ROC: 0.738, p < 0.001, 95% CI: 0.529-0.809, cut-off value 989.3 pg/ml) (**Figure 3A**). By a multiple logistic regression model that included GDF-15 and controlled for the other important clinical parameters, GDF-15 (> 989.3 pg/ml) is a significant predictor for AE occurrence in IPF subjects (p = 0.04) (**Table 3**).

**Table 3:** Risk factors for AE by logistic regression analysis in patients with IPF.

Clinical Variables	Univariate Logistic Analysis			Multivariate Logistic Analysis		
	HR	95.0% CI	p-value	HR	95.0% CI	p-value
Prior corticosteroid use	3.673	1.605-8.404	0.002	5.236	0.799-34.326	0.084
PaO_2_/FiO_2_ (<300)	0.011	0.003-0.043	<0.001	0.021	0.004-0.106	<0.001
GAP stage (I/II/III)	5.227	1.771-15.422	0.003	2.435	0.798-7.542	0.117
Serum GDF-15 (>989.3 pg/ml)	0.246	0.110-0.553	0.001	0.148	0.024-0.914	0.040

### Serum GDF-15 Can Predict the Survival of IPF Patients

The clinical outcomes (including duration in hospital, in-hospital mortality, and total survival) of AE-IPF patients were significantly worse than S-IPF cases. The duration in the hospital of AE-IPF cases was significantly longer (13.36 ± 8.76 vs. 6.72 ± 2.87 days, p < 0.001) and in-hospital mortality was significantly greater than S-IPF subjects (51.61% vs. 0, p < 0.001) (**Table 4**). The Kaplan–Meier curve showed that AE-IPF cases had a significantly higher mortality than S-IPF subjects (p < 0.001) (**Figure 3B**). The serum GDF-15 level was significantly elevated in decedents (n = 47) than that in survivors (n = 51) (p = 0.005) (**Figure 3C**). The areas under the ROC curve of GDF-15 were statistically significant in the distinction of the decedent from the survivor (ROC: 0.680, p = 0.002, 95% CI: 0.572-0.787, cut-off value 1,075.76 pg/ml) (**Figure 3D**). Patients with a serum GDF-15 level above 1,075.76 pg/ml had a greater mortality than subjects lower than that (p < 0.001) (**Figure 3E**). In multivariate Cox regression models, a serum GDF-15 level above 1,075.76 pg/ml and PaO_2_/FiO_2_ were the independent predicting factors for total survival in IPF patients (HR = 0.991, 95% CI: 0.986-0.996, p < 0.001 and HR = 0.428, 95% CI: 0.232-0.790, p = 0.007, respectively) (**Table 5**).

**Table 4:** Clinical outcomes in patients with S-IPF and AE-IPF.

	S-IPF group	AE-IPF group	p-value
Duration in hospital (days)	6.7 ± 2.9	13.5 ± 8.8	<0.001
In-hospital mortality	0/61	16/31	<0.001
Survival state (dead/censor)	14/47	36/11	<0.001
Survival time (days)	1,035.5 ± 416.5	411.6 ± 404.9	<0.001

**Table 5:** Predictors for survival by univariate and multivariate Cox regression models in IPF patients.

Clinical Variables	Univariate Cox Model			Multivariate Cox Model		
	HR	95.0% CI	p-value	HR	95.0% CI	p-value
Concurrent infection	3.052	1.726-5.398	<0.001	1.461	0.763-2.797	0.253
PaO_2_/FiO_2_ ratio	0.989	0.986-0.993	<0.001	0.991	0.986-0.996	<0.001
CT score	1.664	1.400-1.978	<0.001	1.115	0.868-1.432	0.396
Serum GDF-15 (>1,075.76 pg/ml)	0.341	0.195-0.598	<0.001	0.428	0.232-0.790	0.007

## Discussion

GDF-15 has been indicated in various biological functions, including inflammation, metabolism, cancer cachexia, solid tumor, renal and heart failure, atherosclerosis, and lung fibrosis (11, 23, 24). In our study, the differences of clinical parameters associated with infection and inflammation, metabolism, disease severity, and clinical outcomes were significant between patients with AE-IPF and S-IPF. The expression of GDF-15 was increased in AE-IPF cases and the serum GDF-15 level correlated with the clinical variables of inflammation, metabolism, disease severity, and survival of IPF subjects. GDF-15 could be a potential predictor for AE occurrence and survival in IPF patients.

AE is usually a devastating complication for IPF patients (2). Although the tyrosine kinase inhibitor can decrease the incidence of AE, there is still no optimal therapy for AE-IPF (2, 25). The etiology of AE-IPF remains uncertain. Infection, micro-aspiration, and intrinsic biological dysfunction of the lung have been identified as associated with AE in IPF patients (2, 26–29). Our study showed that the clinical parameters related to infection and inflammation (WBC counts, CRP, ESR, and LDH), body metabolism (TG, Apo AI, AIB, and GLU), and disease severity (D-dimer, PaO_2_/FiO_2_ ratio, HRCT score, PAH, FVC, DLCO, GAP scores, and stages) were significantly different between patients with S-IPF and AE-IPF. The published data showed that poor pulmonary physiological function, mechanical procedures, and pulmonary hypertension were associated with AE-IPF (30, 31). So, the development of AE may be related to inflammation and worse pulmonary physiological function, which can cause metabolic disorders. All the above conditions may lead to a vicious cycle and AE occurrence in IPF patients.

Our previous studies indicated that leptin and osteopontin may be the valuable biomarkers for AE-IPF patients (19, 32). However, whether these factors are involved in the development of AE-IPF needs further study in the future. GDF-15 is secreted from senescent AECs and is an aging-related biomarker of IPF (11, 13, 15). Data showed that it might act as a profibrotic risk factor *via* the activation of M2 macrophages and fibroblasts (11). The elevated serum levels of GDF-15 in COVID-19 patients with ARDS indicated that GDF-15 may be associated with ARDS caused by a virus infection (16, 17). The respiratory infection is closely related to the development of AE for IPF patients. Therefore, GDF-15 may be an important trigger factor in the pathogenesis of AE-IPF. GDF-15 serum levels and expression in the lung tissues of AE-IPF patients were significantly elevated in the current study. The serum leptin level in AE-IPF cases was similar to plasma leptin concentrations in our previous study (19). The published studies showed that the inflammatory cytokines of GDF-15 and leptin were associated with infections, metabolism, and lung fibrosis (33–36). Although both serum levels were all climbed up in the serum of AE-IPF subjects, GDF-15 concentrations have a reverse correlation with leptin in this study. The findings showed that both cytokines may be involved in the development of AE in pulmonary fibrosis, but they play a role in the different stages of AE. Furthermore, Lambrecht et al. suggested that GDF-15 expression was induced during fibrosis development and it may participate in fibrosis initiation, but was not indispensable in the course of fibrosis development *in vivo* (37). We suppose that GDF-15 may be involved in the initiation stage of AE and fibrosis.

GDF-15 is closely related to pulmonary inflammation and energy metabolism (10, 12, 16, 38). Wu et al. demonstrated that the overproduction of GDF-15 promoted human rhinovirus 2–induced inflammation in the lung of mice (12). Notz et al. showed that GDF-15 levels were elevated in COVID-19 patients (16). In mice, the overexpression of GDF-15 led to reduced food intake and rising energy expenditure (39). In our cohort study of IPF patients, GDF-15 serum levels correlated positively with the clinical variables of infection and inflammation and negatively correlated with several metabolic indicators. Furthermore, serum GDF-15 levels correlated with multiple parameters of disease severity. Studies showed that GDF-15 plays multiple roles in various pathologies. GDF-15 showed its broad anti-inflammatory function in the animal models of myocardial infarction, atherosclerosis and rheumatoid arthritis. However, it was also identified as a pro-inflammatory cytokine in vascular injury (40, 41) and antiphospholipid syndrome (APS) (42). Hence, GDF-15 may play anti-inflammatory or pro-inflammatory roles in different contexts. The ample evidence above indicates that lung infection and inflammation may lead to the onset of AE and upregulation of GDF-15 in IPF subjects, which can further cause metabolic disorders of lipid and protein and coagulation dysfunction and then aggravate the conditions of these patients. Moreover, these factors may interact with each other and make the clinical conditions of these patients continuously worse under poor pulmonary physiological function.

Serum GDF-15 levels in AE-IPF subjects were not related to smoking in the current study. The published data showed that GDF-15 levels were higher in COPD patients and cigarette smoke (CS)–exposed mice, and increased GDF-15 expression may contribute to CS-induced pulmonary inflammation (43). GDF-15 was directly involved in the production of proinflammatory cytokines and chemokines, such as IL-6 and CCL2 in bleomycin-induced mice and systemic sclerosis (SSc) patients with lung involvement (37). The elevated GDF-15 expression in the lung of IPF patients may trigger inflammation and aggravate disease development (37). Our study demonstrated that serum GDF-15 levels above 989.3 pg/ml can predict AE occurrence in IPF patients. The findings of current and published studies support that the elevated expressions of GDF-15 may be associated with the development of AE by regulating the inflammatory response in IPF patients.

The circulating GDF-15 levels have a close correlation with known markers of fibrosis such as type I collagen and osteopontin (37). GDF-15 expression was induced during fibrosis development and markedly associated with reduced lung function in SSc patients (37). GDF-15 could be a useful biomarker for IPF patients with poor outcomes (13). The short- and long-term clinical outcomes of AE-IPF subjects were all significantly worse than S-IPF cases in our study, consistent with the previous reports (19, 32). ROC curves also demonstrated that serum GDF-15 can predict the survival of IPF patients. The patients with serum GDF-15 levels above 1,075.76 pg/ml had significantly worse survival, and it was an independent risk factor for survival after controlling for other known predictors in IPF patients. We suggest that serum GDF-15 may be a promising biomarker for the survival of IPF subjects.

The present study has some limitations. First, the sample was small and all patients were from the same center. We have no data of serum GDF-15 concentrations from the same patient in different clinical conditions (such as before, during, and after AE), which would be helpful to further support the utility of GDF-15 as a biomarker of IPF. A prospective and multicenter study of a larger sample cohort would be beneficial to provide additional evidence on GDF-15.

## Conclusions

In summary, the expressions of GDF-15 were increased significantly in patients with AE-IPF. An elevated serum GDF-15 level was associated with infection and inflammation, metabolism indicators, and the disease severity of IPF and could predict AE occurrence and the survival in IPF subjects.

## Acknowledgments

The authors would like to thank all patients for sending their consents. This work has been presented in the form of one late-breaking abstract at the European Respiratory Society Congress 2019 in Madrid.

## Data Availability Statement

The original contributions presented in the study are included in the article/**Supplementary Material**. Further inquiries can be directed to the corresponding authors.

## Ethics Statement

The studies involving human participants were reviewed and approved by Ethics Committee of Nanjing Drum Tower Hospital. The patients/participants provided their written informed consent to participate in this study. Written informed consent was obtained from the individual(s) for the publication of any potentially identifiable images or data included in this article.

## Author Contributions 

MC and LS conceived and prepared the manuscript. MC, LS, LinG, ML, YuZ, and LilG contributed to the collection of clinical data, statistics, and experiments. FM and MC evaluated the histopathology. FM and JZ provided the lung tissue sample. MC, XQ, XG, MM, YT, XL, and YX contributed to the administration to the patients. LS, MC, FM, and YX reviewed the manuscript and took responsibility for the integrity and accuracy of data analysis. All authors reviewed and approved the final draft.

## Funding

This study was partly supported by National Natural Science Foundation of China (82070064, 81670059 and 81200049).

## Conflict of Interest

The authors declare that the research was conducted in the absence of any commercial or financial relationships that could be construed as a potential conflict of interest.

## Publisher’s Note

All claims expressed in this article are solely those of the authors and do not necessarily represent those of their affiliated organizations, or those of the publisher, the editors and the reviewers. Any product that may be evaluated in this article, or claim that may be made by its manufacturer, is not guaranteed or endorsed by the publisher.

## Supplementary Materials

Supplementary Figure 1 **(A)** The serum levels of leptin were significantly greater in AE-IPF patients when compared with HCs and S-IPF cases (p *<* 0.001 and p *=* 0.042, respectively). Serum l eptin concentrations were also increased significantly in S-IPF patients compared with HCs (p *<* 0.001). **(B–D)** Serum GDF-15 levels were significantly negatively related to leptin in AE-IPF patients (*r* = - 0.329, p *=* 0.024), but not in S-IPF subjects (*r* = 0.180, p *=* 0.168) or NC cases (r = - 0.345, p = 0.062).

Supplementary Table 1 Baseline clinical characteristics in the three groups.
