# Supplementary figures and images for: Elevated Expression of Growth Differentiation Factor-15 Is Associated With Acute Exacerbation of Idiopathic Pulmonary Fibrosis
Source: Front Immunol. 2022 Jun 15;13:891448. doi: 10.3389/fimmu.2022.891448 (PMC9241490; doi:10.3389/fimmu.2022.891448)

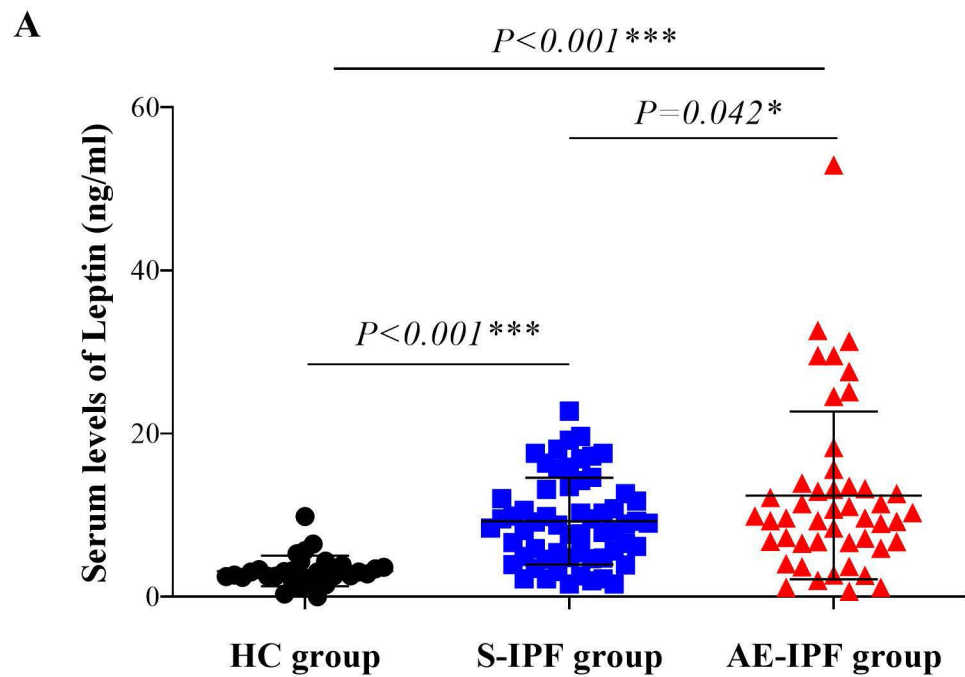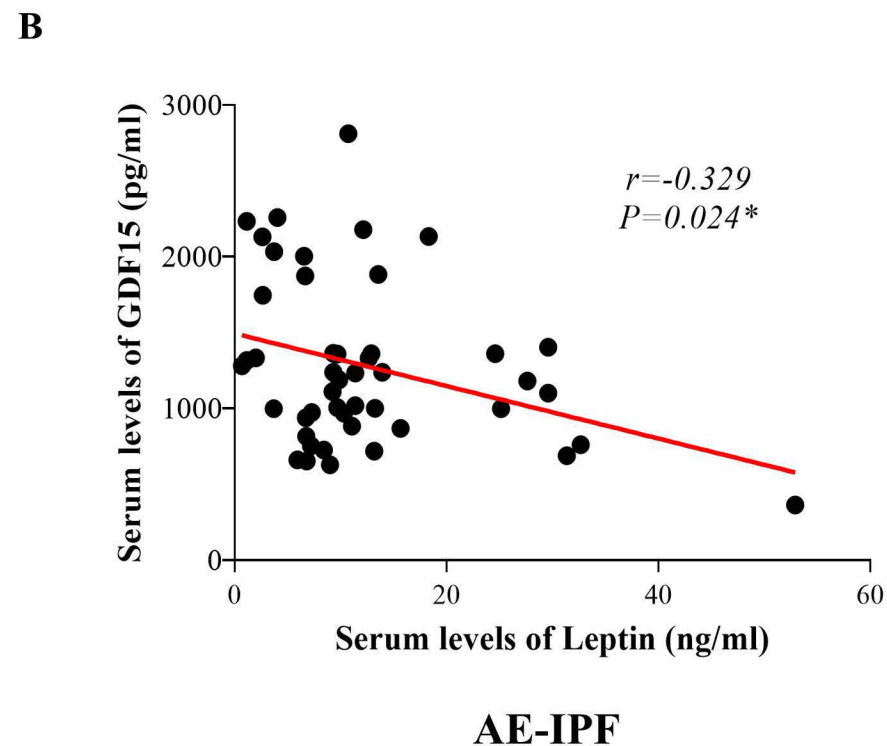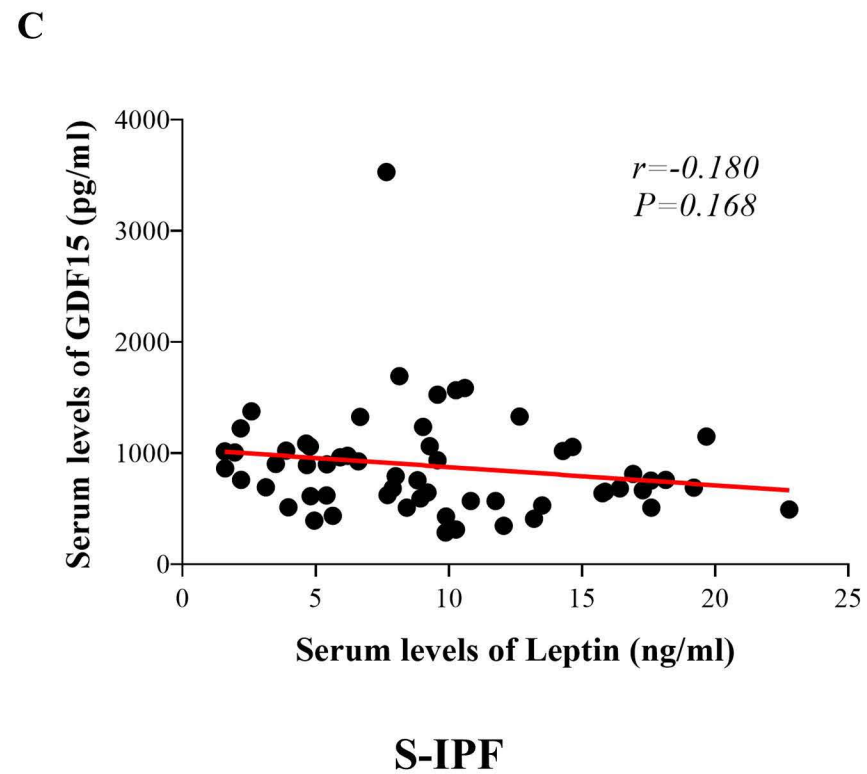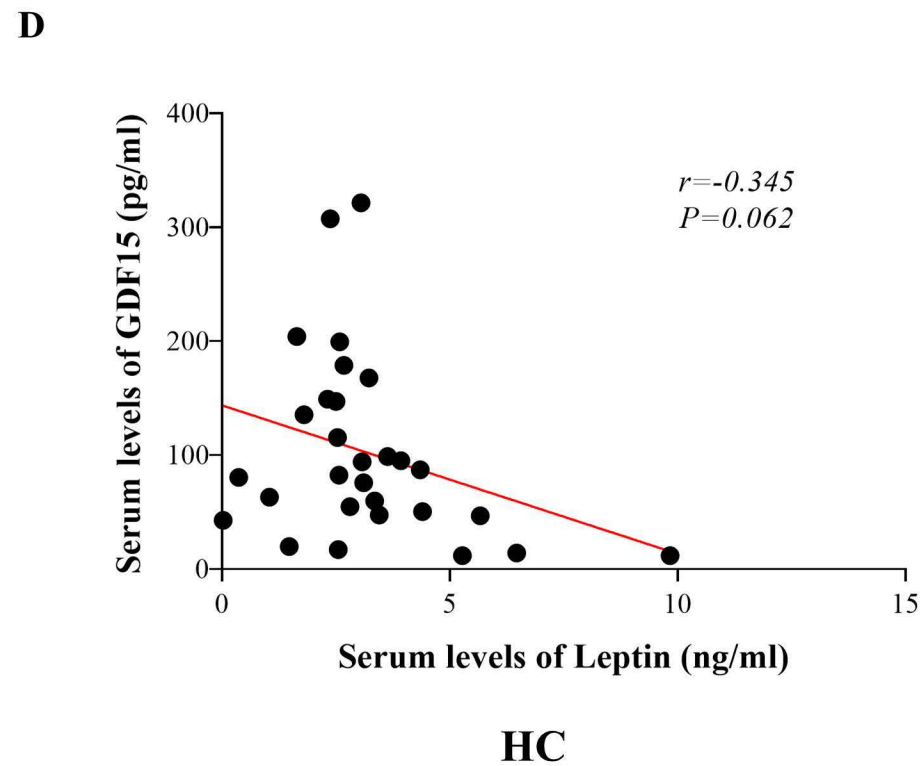

Supplement: Supplementary Figure 1 — (A) The serum levels of leptin were significantly greater in AE-IPF patients when compared with HCs and S-IPF cases (p < 0.001 and p = 0.042, respectively). Serum l eptin concentrations were also increased significantly in S-IPF patients compared with HCs (p < 0.001). (B–D) Serum GDF-15 levels were significantly negatively related to leptin in AE-IPF patients (r = - 0.329, p = 0.024), but not in S-IPF subjects (r = 0.180, p = 0.168) or NC cases (r = - 0.345, p = 0.062). [file Image_1.pdf]
