# Supplementary material for: Elevated Expression of Growth Differentiation Factor-15 Is Associated With Acute Exacerbation of Idiopathic Pulmonary Fibrosis
Source: Front Immunol. 2022 Jun 15;13:891448. doi: 10.3389/fimmu.2022.891448 (PMC9241490; doi:10.3389/fimmu.2022.891448)
Supplement: Supplementary Table 1 — Baseline clinical characteristics in the three groups. [file Table_1.pdf]

Supplementary Data

Suppl. Table 1: Baseline clinical characteristics in three groups of HC, S-IPF and AE-IPF.

	**HC group**	**S-IPF group**	**AE-IPF group**	***P* value**
Gender (M/F)	23/8	49/12	37/10	0.794
Age (years old)	66.16±9.11	66.97±8.33	68.53±7.24	0.085
Complications (Y/N)	-	27/34	25/22	0.438
Prior Antifibrotics use (Y/N)	-	12/49	6/41	0.438
GDF15 (pg/ml)	107.82±14.21	891.30±479.90	1279.22±540.02	<0.001
Leptin (ng/ml)	3.18±1.88	9.15±5.41	12.44±10.27	<0.001
WBC (*10^9)	5.83±1.71	6.56±1.98	10.25±3.72	<0.001
TG (mmol/L)	1.07±0.45	1.56±1.12	1.09±0.85	0.003
TCHL (mmol/L)	4.67±0.61	4.36±0.93	4.35±1.01	0.014
HCHL (mmol/L)	1.53±0.45	1.19±0.60	1.14±0.55	0.853
LCHL (mmol/L)	2.65±0.56	2.59±0.68	2.56±0.85	0.022
Apo AI (g/L)	1.36±0.25	1.10±0.30	0.97±0.28	0.899
Apo B (g/L)	0.84±0.13	0.85±0.21	0.91±0.22	0.282
AIB (g/L)	46.85±2.89	39.36±4.09	33.13±5.58	0.451
GLU (mmol/L)	5.50±1.00	5.34±1.77	6.75±2.14	<0.001
